# Supplementary material for: Long COVID burden and risk factors in 10 UK longitudinal studies and electronic health records
Source: Nat Commun. 2022 Jun 28;13:3528. doi: 10.1038/s41467-022-30836-0 (PMC9240035; doi:10.1038/s41467-022-30836-0)
Supplement: Supplementary file 3 — Description of Additional Supplementary Files [file 41467_2022_30836_MOESM3_ESM.pdf]

Description of Additional Supplementary Files

File Name: Supplementary Data 1
Description: Sources: ALSPAC G1 (Children of the Avon Longitudinal Study of Parents and Children); TwinsUK (UK Adult Twin Registry); ALSPAC G0 (parents of ALSPAC).
