# Supplementary material for: Long COVID burden and risk factors in 10 UK longitudinal studies and electronic health records
Source: Nat Commun. 2022 Jun 28;13:3528. doi: 10.1038/s41467-022-30836-0 (PMC9240035; doi:10.1038/s41467-022-30836-0)
Supplement: Supplementary file 5 — Reporting Summary [file 41467_2022_30836_MOESM5_ESM.pdf]

nature portfolio

Corresponding author(s): Claire J. Steves, Ellen J. Thompson, Dylan M. Williams

Last updated by author(s): Mar 28, 2022

Reporting Summary

Nature Portfolio wishes to improve the reproducibility of the work that we publish. This form provides structure for consistency and transparency in reporting. For further information on Nature Portfolio policies, see our Editorial Policies and the Editorial Policy Checklist.

Statistics

For all statistical analyses, confirm that the following items are present in the figure legend, table legend, main text, or Methods section.

n/a	Confirmed	
☐	☒	The exact sample size (n) for each experimental group/condition, given as a discrete number and unit of measurement
☐	☒	A statement on whether measurements were taken from distinct samples or whether the same sample was measured repeatedly
☐	☒	The statistical test(s) used AND whether they are one- or two-sided<br>*Only common tests should be described solely by name; describe more complex techniques in the Methods section.*
☐	☒	A description of all covariates tested
☐	☒	A description of any assumptions or corrections, such as tests of normality and adjustment for multiple comparisons
☐	☒	A full description of the statistical parameters including central tendency (e.g. means) or other basic estimates (e.g. regression coefficient) AND variation (e.g. standard deviation) or associated estimates of uncertainty (e.g. confidence intervals)
☐	☒	For null hypothesis testing, the test statistic (e.g. F, t, r) with confidence intervals, effect sizes, degrees of freedom and P value noted<br>*Give P values as exact values whenever suitable.*
☒	☐	For Bayesian analysis, information on the choice of priors and Markov chain Monte Carlo settings
☒	☐	For hierarchical and complex designs, identification of the appropriate level for tests and full reporting of outcomes
☐	☒	Estimates of effect sizes (e.g. Cohen's d, Pearson's r), indicating how they were calculated

Our web collection on statistics for biologists contains articles on many of the points above.

Software and code

Policy information about availability of computer code

Data collection	Longitudinal Studies: Data were drawn from 10 UK longitudinal Studies (LS) that had conducted surveys before and during the COVID-19 pandemic comprising five homogeneous cohorts: the Millennium Cohort Study (MCS); the Avon Longitudinal Study of Parents and Children (ALSPAC (generation 1, “G1”); Next Steps (NS); the 1970 British Cohort Study (BCS); and the National Child Development Study (NCDS), and five age-heterogeneous samples were included: the Born in Bradford study (BIB); Understanding Society (USOC); Generation Scotland: the Scottish Family Health Study (GS); the parents of the ALSPAC-G1 cohort, whom we refer to as ALSPAC-G0; and the UK Adult Twin Registry (TwinsUK).<br>Electronic Health Records: Data from primary care records managed by the GP software provider TPP SystmOne software was linked to Secondary Uses Service (SUS) data (containing hospital records) through OpenSAFELY (https://www.opensafely.org/).
Data analysis	Analysis code for the meta-analyses and forest plotting of long COVID risk factors from 10 LS samples can be accessed on https://github.com/dylwil/longCOVIDrisk. All code for the OpenSAFELY platform for data management, analysis and secure code execution is shared for review and re-use under open licenses at https://github.com/opensafely. All codelists (describing the definition of the conditions) and the code for data management and analysis is shared for scientific review and re-use under open licenses on GitHub https://github.com/opensafely/long-covid-historical-health, with the code archived on Zenodo (https://doi.org/10.5281/zenodo.6361864).

For manuscripts utilizing custom algorithms or software that are central to the research but not yet described in published literature, software must be made available to editors and reviewers. We strongly encourage code deposition in a community repository (e.g. GitHub). See the Nature Portfolio guidelines for submitting code & software for further information.

Data

Policy information about availability of data

All manuscripts must include a data availability statement. This statement should provide the following information, where applicable:

- Accession codes, unique identifiers, or web links for publicly available datasets
- A description of any restrictions on data availability
- For clinical datasets or third party data, please ensure that the statement adheres to our policy

Data access for NCDS (SN 6137), BCS70 (SN 8547), Next Steps (SN 5545), MCS (SN 8682) and all four COVID-19 surveys (SN 8658) can be obtained through the UK Data Service. ALSPAC data is available to researchers through an online proposal system. Information regarding access can be found on the ALSPAC website (http://www.bristol.ac.uk/media-library/sites/alspac/documents/researchers/data-access/ALSPAC_Access_Policy.pdf). Data from the various Born in Braford family studies are available to researchers; see the study website for information on how to access data (https://borninbradford.nhs.uk/research/how-to-access-data/).
Generation Scotland data are available through the UK Data Service (SN 6614 and SN 8644). Access to data is approved by the Generation Scotland Access Committee. See https://www.ed.ac.uk/generation-scotland/for-researchers/access or email access@generationscotland.org for further details.
TwinsUK data are available on request from the TwinsUK Resource Executive Committee (TREC). Access to TwinsUK data can be obtained via a standard application procedure. Data requests should be submitted via https://twinsuk.ac.uk/resources-for-researchers/access-our-data/.

Field-specific reporting

Please select the one below that is the best fit for your research. If you are not sure, read the appropriate sections before making your selection.

☐ Life sciences ☒ Behavioural & social sciences ☐ Ecological, evolutionary & environmental sciences

For a reference copy of the document with all sections, see nature.com/documents/nr-reporting-summary-flat.pdf

Behavioural & social sciences study design

All studies must disclose on these points even when the disclosure is negative.

Study description	The UK National Core Studies – Longitudinal Health and Wellbeing programme (https://www.ucl.ac.uk/covid-19-longitudinal-health-wellbeing/) combines data from multiple UK population-based LS and electronic health records (EHR) to answer pandemic-relevant questions. In this analysis we pooled results from parallel analyses within individual LS, then compared with population-based findings from EHR capturing individuals who actively sought healthcare.
Research sample	Longitudinal Studies: Data were drawn from 10 UK longitudinal Studies (LS) that had conducted surveys before and during the COVID-19 pandemic comprising five homogeneous cohorts: the Millennium Cohort Study (MCS); the Avon Longitudinal Study of Parents and Children (ALSPAC (generation 1, "G1"); Next Steps (NS); the 1970 British Cohort Study (BCS); and the National Child Development Study (NCDS), and five age-heterogeneous samples were included: the Born in Bradford study (BIB); Understanding Society (USOC); Generation Scotland: the Scottish Family Health Study (GS); the parents of the ALSPAC-G1 cohort, whom we refer to as ALSPAC-G0; and the UK Adult Twin Registry (TwinsUK).<br>Electronic Health Records: Working on behalf of NHS England, we conducted a population-based cohort study to measure long COVID recording in electronic health record (EHR) data from primary care practices using TPP SystmOne software, linked to Secondary Uses Service (SUS) data (containing hospital records) through OpenSAFELY (https://www.opensafely.org/).
Sampling strategy	Longitudinal Studies: Data were drawn from 10 longitudinal UK population studies which conducted surveys both before and during the COVID-19 pandemic.
Data collection	Longitudinal Studies: COVID-19 cases were defined by self-report, including testing confirmation and health care professional diagnosis.<br>Electronic Health Records: From a population of all people alive and registered with a general practice on 1st December 2020, we selected all patients who had evidence of a COVID-19 related code, either: positive SARS-CoV-2 testing, being hospitalised with an associated COVID diagnostic code, or having a recorded diagnostic code for COVID in primary care.
Timing	Longitudinal Studies: All data used to derive these outcomes were collected between April-November 2020.<br>Electronic Health Records: The outcome was measured between the study start date (1st February 2020) and the end date (9th May 2021).
Data exclusions	Longitudinal Studies: Minimum inclusion criteria were pre-pandemic health measures, age, sex, ethnicity plus self-reported COVID-19, and self-reported duration of COVID-19 symptoms.
Non-participation	NA
Randomization	Longitudinal Studies: A minimal set of covariates were adjusted for across all studies, where relevant: age (adjusted as a continuous variable when being considered a covariate), sex, and ethnicity.

Reporting for specific materials, systems and methods

We require information from authors about some types of materials, experimental systems and methods used in many studies. Here, indicate whether each material, system or method listed is relevant to your study. If you are not sure if a list item applies to your research, read the appropriate section before selecting a response.

Materials & experimental systems

n/a	Involved in the study
☒	☐ Antibodies
☒	☐ Eukaryotic cell lines
☒	☐ Palaeontology and archaeology
☒	☐ Animals and other organisms
☐	☒ Human research participants
☒	☐ Clinical data
☒	☐ Dual use research of concern

Methods

n/a	Involved in the study
☒	☐ ChIP-seq
☒	☐ Flow cytometry
☒	☐ MRI-based neuroimaging

Human research participants

Policy information about studies involving human research participants

Population characteristics	See above In total, there were 48,901 individuals across 10 UK longitudinal studies
Recruitment	Data were drawn from 10 longitudinal UK population studies which conducted surveys both before and during the COVID-19 pandemic.
Ethics oversight	The most recent sweeps of the NCDS, BCS70, Next Steps and MCS have all been granted ethical approval by the National Health Service (NHS) Research Ethics Committee and all participants have given informed consent. Ethical approval was obtained from the ALSPAC Ethics and Law Committee and the Local Research Ethics Committees. Ethical approval for Born in Bradford was granted by the National Health Service Health Research Authority Yorkshire and the Humber (Bradford Leeds) Research Ethics Committee (reference: 16/YH/0320). The University of Essex Ethics Committee has approved all data collection for the Understanding Society main study and COVID-19 waves. Generation Scotland obtained ethical approval from the East of Scotland Committee on Medical Research Ethics (on behalf of the National Health Service). All wave of TwinsUK have received ethical approval associated with TwinsUK Biobank (19/NW/0187), TwinsUK (EC04/015) or Healthy Ageing Twin Study (H.A.T.S) (07/H0802/84) studies from NHS Research Ethics Committees at the Department of Twin Research and Genetic Epidemiology, King's College London.

Note that full information on the approval of the study protocol must also be provided in the manuscript.
